# Exercise reshapes gut microbiota to ameliorate core symptoms in PCOS: molecular mechanisms and therapeutic implications

**DOI:** 10.3389/fendo.2025.1652731

**Published:** 2025-10-29

**Authors:** Qianqian Li, Leqin Chen, Ronghui Wang

**Affiliations:** ^1^ Sport Science School, Beijing Sport University, Beijing, China; ^2^ School of Physical Education, Shanxi Normal University, Taiyuan, China

**Keywords:** pcos, gut microbiota, exercise, molecular mechanisms, IR

## Abstract

**Background:**

Polycystic ovary syndrome (PCOS) is a prevalent endocrine-metabolic disorder characterized by Insulin Resistance (IR), hyperandrogenism, and ovulatory dysfunction, with gut dysbiosis emerging as a key pathophysiological driver. Exercise, a non-pharmacological intervention, ameliorates PCOS symptoms, yet the molecular mechanisms linking exercise-induced gut microbiota remodeling to metabolic improvements remain elusive.

**Objective:**

This review synthesizes evidence on how exercise reshapes gut microbiota to reverse core PCOS pathologies through integrated molecular pathways.

**Results:**

Exercise enriches beneficial taxa (e.g., *Faecalibacterium*, *Roseburia*, *Akkermansia muciniphila*) and reduces pro-inflammatory pathogens (e.g., Proteobacteria), elevating short-chain fatty acids (SCFAs) and secondary bile acids (BAs) while suppressing lipopolysaccharide (LPS) translocation. We propose three core mechanisms:(1) SCFAs network reconstruction: Butyrate/propionate enhance gut barrier integrity (via ZO-1/Occludin), inhibit histone deacetylases (suppressing CYP17A1), activate GLP-1 secretion (FFAR3-dependent), and mitigate inflammation. (2) BA-FXR axis activation: Exercise increases secondary BAs (e.g., deoxycholic acid), activating hepatic FXR to inhibit gluconeogenesis (*PEPCK/G6Pase*) and upregulate androgen-clearance enzymes (*SULT2A1/CYP3A4*). (3) LPS-inflammation inhibition: Reduced LPS blunts TLR4/NF-κB signaling and NLRP3 inflammasome activation, resolving chronic inflammation. These axes converge to improve tissue-specific PCOS features: ovarian androgen synthesis (HDAC/NF-κB inhibition), hepatic IR (FXR/PI3K-Akt), and ovulatory function (AhR-mediated Treg/Th17 balance). Exercise modality differentially impacts PCOS subtypes—endurance training benefits IR-dominant phenotypes via SCFAs producers, while resistance training reduces inflammation in obese PCOS.

**Conclusion:**

Exercise remodels the gut microbiota-metabolism-immune network to reverse PCOS pathophysiology. Targeting microbial metabolites (e.g., butyrate, BAs) or their receptors (FXR, GPR43) offers novel therapeutic strategies. Future research must address PCOS heterogeneity and optimize exercise protocols for microbiota-directed precision medicine.

## Highlights

- Exercise enriches SCFA-producers (*Faecalibacterium*, *Roseburia*) and *Akkermansia* while reducing Proteobacteria.
- SCFAs activate FFAR3/GPR43 to enhance GLP-1 secretion, gut barrier integrity, and HDAC inhibition.
- Secondary bile acids activate FXR to suppress hepatic gluconeogenesis and upregulate androgen clearance.
- Exercise inhibits LPS-TLR4/NF-κB and NLRP3 inflammasome, resolving chronic inflammation in PCOS.
- Modality-specific effects: Endurance training targets IR; resistance training reduces inflammation in obese PCOS.

## 1 Introduction

Polycystic Ovary Syndrome (PCOS) is a prevalent endocrine-metabolic disorder affecting millions of women of reproductive age (1, 2). Its core features include metabolic dysfunction, notably insulin resistance (IR), hormonal imbalances such as hyperandrogenism, and chronic low-grade inflammation. These disruptions contribute to significant health risks, including infertility, type 2 diabetes, and cardiovascular disease (1, 3–6). Global prevalence estimates range from 8% to 18%, varying significantly with diagnostic criteria and study populations (7). The etiology of PCOS is multifactorial, involving genetic, environmental, and lifestyle factors. Notably, gut microbiota dysbiosis has emerged as a key pathophysiological driver, exacerbating inflammation, oxidative stress, and metabolic abnormalities central to the syndrome (8, 9).

The gut microbiota, a complex ecosystem integral to host metabolism and immunity (10, 11), can directly modulate pathways involved in PCOS pathogenesis, including steroid hormone metabolism and insulin sensitivity (12). Accumulating evidence confirms that gut dysbiosis is a hallmark of PCOS and is mechanistically linked to its development and progression (8, 9).

Current PCOS treatments, while effective, often carry risks of adverse effects, highlighting the need for safer alternatives. Exercise, a non-pharmacological intervention, has demonstrated efficacy in alleviating PCOS symptoms (13, 14). However, the precise molecular mechanisms through which exercise-induced gut microbiota remodeling confers these metabolic benefits remain incompletely defined.

A recent comprehensive review has extensively summarized the role of gut microbiota in the pathogenesis and metabolic disorders of PCOS, including insulin resistance, hormonal imbalances, bile acid metabolism dysregulation, IL-22-mediated immune dysregulation, and brain-gut axis disturbances (15). Furthermore, as a non-pharmacological intervention, exercise has been demonstrated to significantly alter the composition and function of the gut microbiota, suggesting a novel mechanistic pathway through which it may ameliorate PCOS symptoms. While that review provides a broad overview of microbial contributions to PCOS, the present review distinctively focuses on the mechanistic links between exercise-induced gut microbiota remodeling and the amelioration of core PCOS symptoms.Therefore, this review aims to systematically integrate the current knowledge on the complex interrelationships among exercise intervention, gut microbiota composition/function (including microbial metabolites), and key PCOS pathological features, with a specific focus on the underlying molecular mechanisms driving metaboic remodeling. Our primary objective is to elucidate the specific molecular pathways through which exercise modulates the gut microbiome to improve metabolic homeostasis (notably insulin sensitivity), reduce hyperandrogenism, and mitigate inflammation in PCOS. By establishing a unified mechanistic framework linking exercise-induced microbial shifts to metabolic and endocrine improvements, this review seeks to fill a critical knowledge gap and provide robust scientific evidence essential for developing novel, effective, and microbiota-targeted therapeutic strategies, including optimized exercise prescriptions, for the management of PCOS.

## 2 The pathological association between gut dysbiosis and PCOS

### 2.1 Gut microbiota characteristics in PCOS patients

Current research on gut microbiota diversity in PCOS patients shows conflicting results. While most cross-sectional studies report a significant decrease in α-diversity in PCOS patients compared to healthy individuals (15), a meta-analysis found no statistically significant differences in overall diversity (16). This discrepancy likely arises from the high heterogeneity within the PCOS patient population and inadequate control of confounding factors such as dietary habits and antibiotic use in study designs (17).

Characteristic alterations are observed across multiple taxonomic ranks. At the phylum level, several studies confirm a significant increase in Proteobacteria abundance, particularly Gram-negative bacteria like Escherichia spp. and *Shigella* spp., whose lipopolysaccharides (LPS) may contribute to PCOS pathogenesis by triggering systemic chronic inflammation (18). Additionally, an imbalanced Bacteroidetes to Firmicutes ratio (F/B ratio) is widely reported, potentially exacerbating metabolic dysregulation (17). At the genus/species level, there is enrichment of opportunistic pathogens (e.g., *Bacteroides* spp., *Escherichia* spp., *Shigella* spp., *Lactobacillus*, Bacteroidaceae, and *Klebsiella* spp.) (19–21) alongside depletion of beneficial bacteria such as *Akkermansia* and *Roseburia* (11). A pronounced reduction is observed in short-chain fatty acid (SCFAs)-producing bacteria, including *Roseburia*, *Faecalibacterium*, and *Bifidobacterium*, whose functional loss may exacerbate PCOS-related metabolic and endocrine abnormalities (16).

Gut microbiota composition also differs according to PCOS phenotype. Obese PCOS patients exhibit increased Enterobacteriaceae and reduced *Lactobacillus* and *Bifidobacterium* compared to non-obese PCOS patients and healthy controls, changes associated with elevated inflammation and IR (22). Differences in gut microbiota composition and structure are also noted between PCOS patients with and without IR (23). These phenotype-specific microbial patterns highlight the need for targeted interventions that can modulate the gut ecosystem.

These characteristic shifts in gut microbiota composition (**Table 1**), including the reduction in SCFAs-producing bacteria and the increase in pro-inflammatory taxa, have been suggested to play a significant role in driving the metabolic dysfunction central to PCOS pathology.

**Table 1:** Proposed functional link to PCOS pathology.

Classification level	PCOS Microbiome changes	Key species	Function/phenotype association	Evidence sources
Phylum	↑ Firmicutes/Bacteroidetes ratio	Escherichia coli, Shigella	Increased LPS, inflammation	(18, 21)
	Altered F/B ratio (direction inconsistent)		Energy acquisition, metabolic dysregulation	(16, 17)
Genus/Species	↑ Opportunistic pathogens	*Bacteroides*, Escherichia, Shigella, *Lactobacillus* (partial studies), Bacteroidaceae, Klebsiella	Inflammation, barrier dysfunction	(19–21)
	↓ Beneficial SCFAs producers	*Akkermansia*, *Roseburia*, *Faecalibacterium*, *Bifidobacterium*	Reduced SCFAs (butyrate, acetate, propionate), impaired metabolic and immune regulation	(9, 16)
Phenotypic Differences	Obese PCOS vs non-obese PCOS/control group	↑ Enterobacteria, ↓ *Lactobacillus*, ↓ *Bifidobacterium* (Obese PCOS)	Associated with higher inflammation and IR	(22)
	Insulin-resistant PCOS vs non-insulin-resistant PCOS	Different composition/structure	Specific association with I mechanisms	(23)

F/B ratio refers to the ratio of Firmicutes to Bacteroidetes; SCFA refers to SCFAs; LPS refers to lipopolysaccharides; IR refers to IR. Diversity results (α-diversity) show inconsistencies across different studies (15, 16).

### 2.2 Hyperandrogenism

Hyperandrogenism, a core pathological feature of PCOS, is linked to gut dysbiosis through a complex interaction network involving inflammation, epigenetic regulation, bile acid metabolism, and neuroendocrine interactions.

Chronic inflammation serves as a central mechanism linking gut dysbiosis to excessive androgen synthesis. Gut barrier disruption facilitates translocation of bacterial metabolites, particularly LPS from Gram-negative bacteria (e.g., Escherichia, Shigella), into systemic circulation (18, 21, 24). Circulating LPS targets the ovaries and activates the TLR4/NF-κB pathway, triggering local inflammatory responses (25). Specifically, (1) LPS activates ovarian NF-κB signaling via the TLR4-MyD88-dependent pathway, directly upregulating key androgen synthesis enzymes (CYP17A1 and CYP11A1) in granulosa cells (26).(2) (2) LPS-induced inflammatory cytokines, particularly IL-6 and TNF-α, synergistically amplify androgen effects - IL-6 activates JAK/STAT or MAPK signaling to induce androgen synthesis enzyme expression (27, 28), while TNF-α inhibits aromatase (CYP19A1) via the NF-κB pathway, blocking androgen-to-estrogen conversion (29). These mechanisms increase androgen production while inhibiting its clearance. Furthermore, chronic inflammation reduces sex hormone-binding globulin (SHBG) synthesis, enhancing free testosterone bioactivity (30).

SCFAs deficiency exacerbates these effects. Depletion of SCFAs-producing bacteria (e.g., *Roseburia*, *Faecalibacterium*) significantly reduces butyrate levels, removing its inhibitory effects on androgen synthesis through multiple pathways: (1) Butyrate deficiency weakens gut barrier function by impairing tight junction protein expression, increasing permeability and LPS translocation (31). (2) (2) As a histone deacetylase (HDAC) inhibitor, butyrate deficiency increases histone H3K9 acetylation in the CYP17A1 promoter region, abnormally activating androgen synthesis (32). (3) SCFAs activate free fatty acid receptors (FFAR2/3), stimulating glucagon-like peptide-1 (GLP-1) secretion - loss of this function exacerbates IR, which further stimulates ovarian androgen secretion (33).

**Figure 1:**
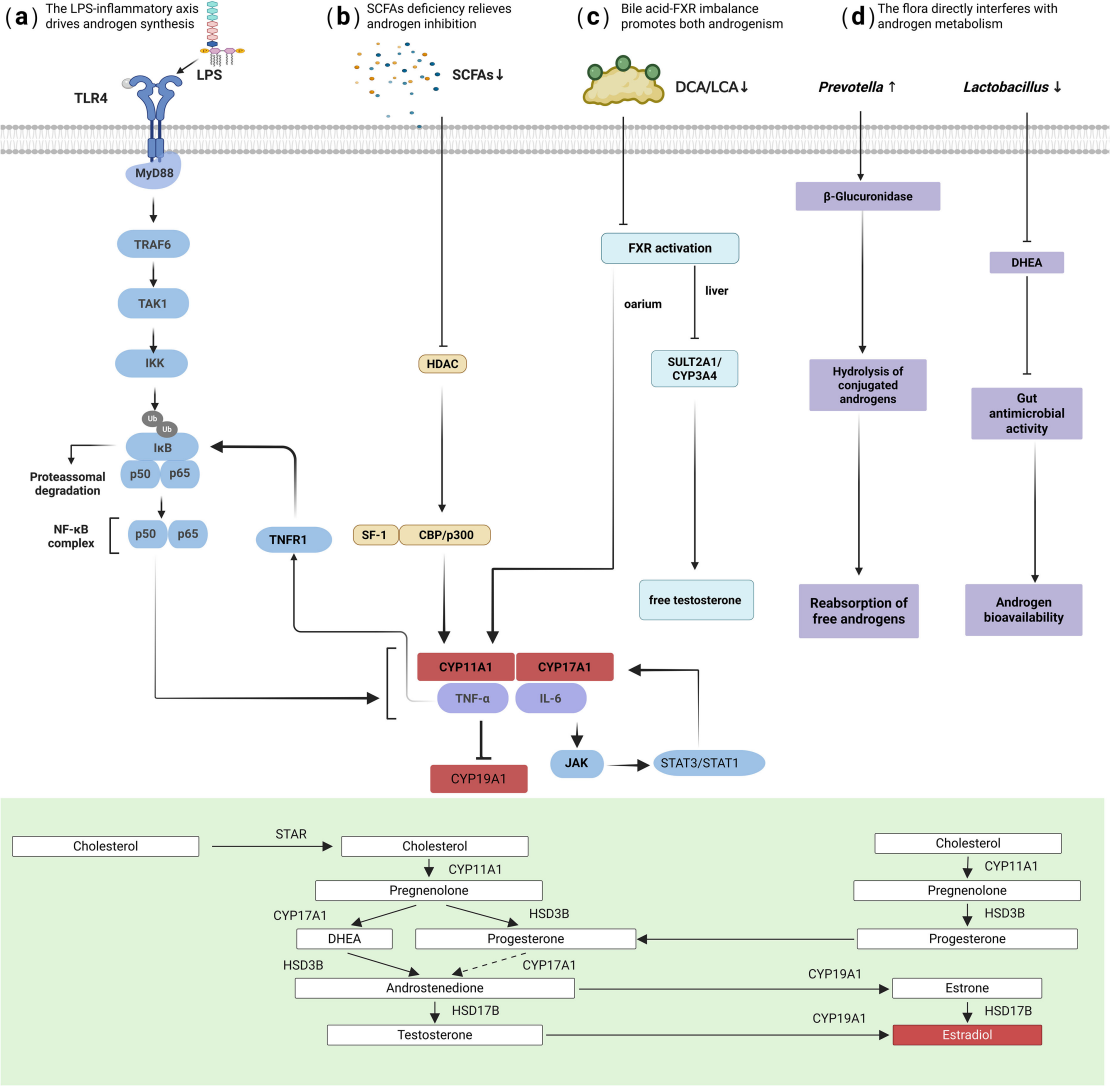
Mechanisms of hyperandrogenism driven by gut dysbiosis. The key pathways illustrated include: **(a)** LPS-TLR4/NF-κB activation: LPS from gut pathogens activates ovarian TLR4/NF-κB signaling, upregulating key androgen-synthesis enzymes (CYP17A1, CYP11A1). **(b)** Inflammatory cytokine signaling: TNF-α and IL-6 inhibit aromatase (CYP19A1) and synergistically amplify androgen synthesis. **(c)** Bile acid-FXR axis imbalance: Reduced secondary bile acids lead to insufficient FXR activation, weakening its inhibition of ovarian CYP17A1 and impairing hepatic androgen clearance. **(d)** Direct microbial enzymatic regulation: Bacterial β-glucuronidase hydrolyzes conjugated androgens into free, active forms.

Bile acid metabolism reprogramming provides another mechanism for microbiota-mediated androgen regulation (34)(**Figure 1c**). Under dysbiosis, conversion of primary bile acids to secondary bile acids (e.g., deoxycholic acid) by bacteria like *Bacteroides* and Clostridia is reduced (35). This leads to insufficient activation of the Farnesoid X receptor (FXR), triggering dual pathological effects: (1) In the liver, loss of negative feedback results in excessive bile acid synthesis (upregulated CYP7A1/CYP27A1) (36). (2) In the ovary, insufficient local FXR activation weakens its inhibition of CYP17A1 transcription, directly promoting testosterone synthesis (36). Additionally, bile acid dysregulation impairs hepatic androgen inactivation (37).

Direct microbial enzymatic modulation also contributes: enrichment of Prevotella may promote hydrolysis of conjugated androgens to free forms via β-glucuronidase activity (**Figure 1d**) (38, 39), while reduction of *Lactobacillus* diminishes its protective effects, including competitive consumption of androgen precursors and intestinal acidification to inhibit pathogens (40).

These multifaceted mechanisms driven by gut dysbiosis not only elevate circulating androgens but also exacerbate underlying metabolic disturbances, creating a vicious cycle that underpins the PCOS phenotype and suggesting that interventions targeting gut microbiota could break this cycle.

### 2.3 Insulin resistance

**Figure 2:**
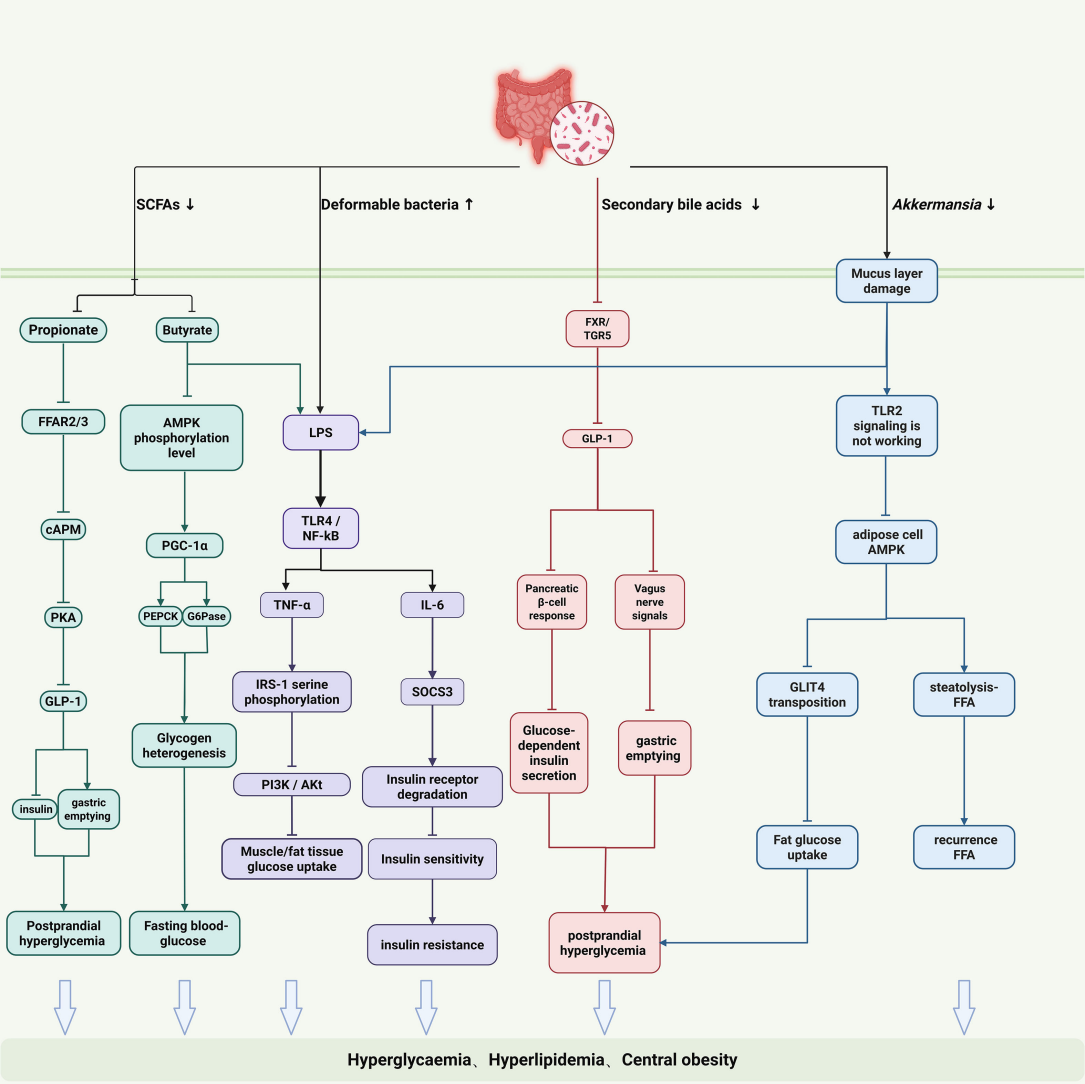
Gut microbiota-driven mechanisms of insulin resistance in PCOS. The illustrated pathways include: (1) SCFA deficiency: Impairs gut barrier, leading to LPS translocation, and reduces key metabolic signals (e.g., GLP-1). (2) LPS-induced inflammation: Circulating LPS and cytokines (TNF-α, IL-6) cause insulin signaling defects via serine phosphorylation of IRS-1 and SOCS3-mediated receptor degradation. (3) BA-FXR/TGR5 dysregulation: Affects glucose and lipid metabolism and gut hormone secretion. (4) Gut-brain axis disruption: Involving Akkermansia, SCFAs, and microbial metabolites, impacts central appetite regulation, fat storage, and energy expenditure.

Gut microbiota dysbiosis drives insulin resistance onset and progression through interconnected pathways. SCFAs deficiency is a core initiating factor. Butyrate deficiency impairs gut barrier integrity (downregulating ZO-1 and Occludin), exacerbates hepatic gluconeogenesis via AMPK signaling deactivation (evidenced by upregulated PEPCK and G6Pase expression), and impairs peripheral glucose uptake. Propionate deficiency reduces activation of free fatty acid receptors FFAR2/3, diminishing GLP-1 secretion, which leads to insufficient insulin secretion and accelerated gastric emptying, disrupting glucose homeostasis (41). Furthermore, SCFAs deficiency-induced barrier damage promotes LPS translocation into the bloodstream, triggering chronic inflammation. In this inflammatory state: TNF-α induces serine phosphorylation of IRS-1, blocking PI3K/Akt signaling, while IL-6 upregulates SOCS3 to accelerate insulin receptor degradation, establishing a “metabolic-inflammation” vicious cycle (**Figure 2**).

Bile acid-FXR/TGR5 signaling dysregulation exacerbates glucose/lipid imbalance. Dysbiosis hinders microbial conversion of primary to secondary bile acids, leading to insufficient FXR activation (42, 43). Consequences include: (1) Impaired hepatic FXR signaling fails to inhibit CYP7A1 via negative feedback, resulting in excessive bile acid synthesis; (2) Dysfunctional intestinal FXR/TGR5 pathway reduces GLP-1 secretion, aggravating insulin secretion defects (44, 45).

Disrupted gut-brain axis regulation provides a central mechanism for IR. Reduced *Akkermansia muciniphila* abundance damages intestinal mucus layer integrity and diminishes its vesicle-mediated, TLR2-dependent improvement of adipose tissue insulin sensitivity (46). SCFAs deficiency suppresses pro-opiomelanocortin (POMC) neurons in the hypothalamic arcuate nucleus, promoting appetite enhancement and energy excess via neuropeptide Y (NPY) and GLP-1 pathways (47, 48). Microbial metabolites (e.g., secondary bile acids, indole derivatives) modulate sympathetic nervous activity via vagus nerve afferents, impairing brown adipose tissue thermogenesis and energy expenditure (49).This intricate network of peripheral and central mechanisms highlights how gut microbiota dysbiosis orchestrates IR through multiple parallel pathways, suggesting that effective therapeutic strategies must address this complexity.

### 2.4 Ovulatory disorders

**Figure 3:**
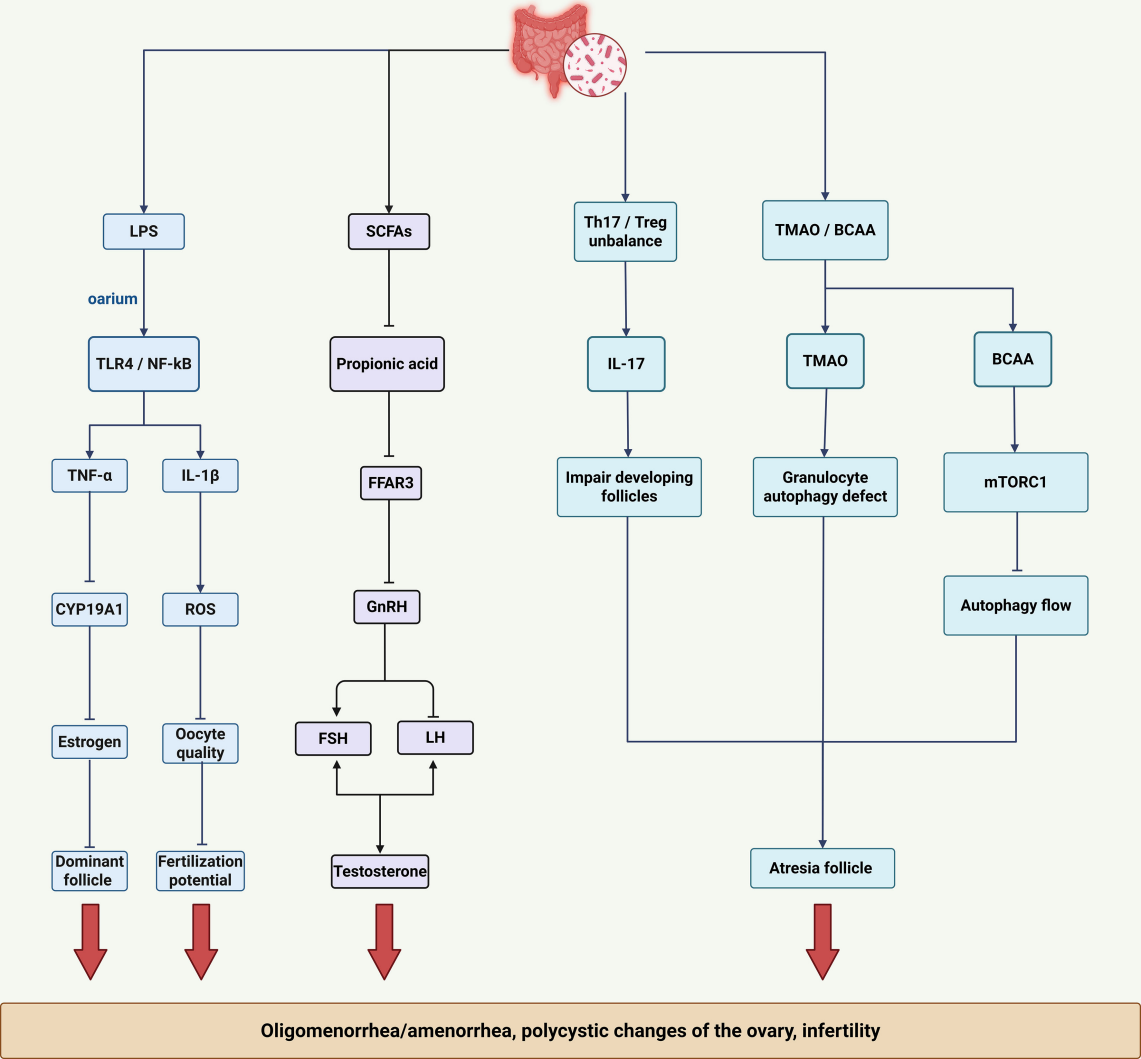
Mechanisms of ovulatory dysfunction mediated by gut dysbiosis. The key factors highlighted are: Inflammatory microenvironment: LPS, TNF-α, and IL-1β impair follicle development and oocyte quality. SCFAs deficiency: Disrupts endocrine rhythms (GnRH pulsatile secretion, LH/FSH ratio) and estrogen response. Immune-metabolic imbalance: Th17/Treg imbalance and cytotoxic metabolites (TMAO, BCAA) directly contribute to follicle atresia through mechanisms such as mitochondrial autophagy defects (TMAO) and inhibition of autophagic flux (BCAA/mTORC1).

Gut microbiota dysbiosis interferes with follicular development and ovulation primarily through inflammatory microenvironment remodeling, endocrine axis disruption, and direct cytotoxic effects. (**Figure 3**). An inflammatory microenvironment particularly impairs folliculogenesis. Gut-derived LPS activates ovarian TLR4/MyD88/NF-κB signaling, causing granulosa cells to secrete pro-inflammatory cytokines such as TNF-α and IL-1β. Specifically, TNF-α inhibits aromatase (CYP19A1), consequently reducing estrogen production, impairing follicle development, and hindering dominant follicle formation. IL-1β further induces mitochondrial reactive oxygen species (ROS) generation, resulting in oocyte developmental abnormalities (50). Additionally, inflammatory processes are synergistically exacerbated by hyperinsulinemia and hyperandrogenism. Elevated insulin levels increase ovarian theca cell sensitivity to IGF-1, enhancing CYP17A1 activity and promoting androgen synthesis (51). Hyperandrogenism subsequently downregulates follicle-stimulating hormone (FSH) receptor expression, inhibiting granulosa cell proliferation and further compromising follicular maturation (51).

Gut microbiota dysbiosis leads to loss of SCFAs, impairing their essential metabolic regulatory functions and disrupting reproductive endocrine homeostasis. Butyrate deficiency impairs histone deacetylase (HDAC) inhibition, leading to dysregulated estrogen receptor (ER) expression and reduced endometrial responsiveness to estrogen (52). Concurrently, propionate deficiency inhibits FFAR3 signaling, disrupting hypothalamic gonadotropin-releasing hormone (GnRH) pulsatility, increasing the LH/FSH ratio, and stimulating premature androgen synthesis in ovarian theca cells.

Gut dysbiosis-related immunometabolic disturbances directly drive follicular atresia. Dysbiosis-induced imbalance between T helper 17 (Th17) and regulatory T (Treg) cells exacerbates follicular damage: elevated Th17 cells produce IL-17, directly damaging developing follicles, while reduced Treg cells diminish follicular protection (53). Furthermore, cytotoxic gut-derived metabolites autophagy defects in granulosa cells, accelerating follicular degeneration. Similarly, accumulation of branched-chain amino acids (BCAAs) activates mTORC1 signaling, suppressing autophagic flux and thereby enhancing follicular damage. The multifactorial disruption of ovulatory function by gut dysbiosis is intrinsically linked to accompanying endocrine and metabolic imbalances, further compromising fertility and overall metabolic health in PCOS and identifying multiple potential targets for therapeutic intervention.

## 3 Exercise and gut microbiota

Given the established role of gut microbiota dysbiosis in the pathogenesis of PCOS, investigating the therapeutic potential of exercise is crucial. As a non-pharmacological intervention, exercise demonstrably influences the composition and function of the gut microbiota, suggesting a promising strategy for ameliorating PCOS symptoms. This section examines the mechanisms by which exercise modulates the gut microbiota and its functional consequences.

### 3.1 Animal model evidence

Extensive rodent research indicates that exercise training modifies gut microbiota composition and function (54–68). Key findings in animal models include:

F/B Ratio Variability: Changes in the Firmicutes/Bacteroidetes ratio are common but directionally inconsistent (increase (55, 56, 67), decrease (57–59), or no change (61, 62)), influenced by exercise modality, diet, age, and host genetics. For instance, voluntary wheel running (VWR) increased microbial diversity specifically in high-fat diet-fed mice (57), and juvenile rats showed more pronounced genus-level changes than adults after VWR (60).

Exercise Modality Matters: The type of exercise (voluntary vs. forced) differentially shapes microbial communities (63).

Beneficial Shifts: A consistent finding is the enrichment of beneficial bacteria, particularly SCFAs-producers (e.g., increased butyrate levels (62) and butyrate-producing taxa (57, 67)).

Pathogen Reduction: Exercise can decrease opportunistic pathogens (e.g., *Pseudomonas*, Enterobacteriaceae, *Aeromonas* after resistance training (68)).

### 3.2 Human evidence

Human studies corroborate exercise’s impact on gut microbiota.

Cross-sectional Evidence: Higher fitness levels correlate with greater diversity and abundance of butyrate-producing bacteria (69). Active individuals (e.g., women exercising ≥3h/week, athletes) show enrichment of beneficial taxa (e.g., *Faecalibacterium prausnitzii*, *Roseburia hominis*, *Akkermansia muciniphila*) and elevated fecal SCFAs concentrations compared to sedentary controls (70, 71). Athletes also exhibit distinct microbial metabolic pathways (70).

Longitudinal Interventions: Structured exercise programs induce specific changes. Endurance training (6 weeks, 3x/week) increased *Faecalibacterium* in lean individuals and butyrate producers/SCFAs (lean only), while decreasing them in obese participants where *Bacteroides* increased (72). Cycling (6 weeks) increased A. muciniphila and decreased Proteobacteria (73). An 8-week program increased Firmicutes abundance and specific butyrate producers (Ruminococcus gauvreauii, Firmicutes FCS020, Anaerobiospirillum) (74).

Collectively, evidence from both animal models and human studies demonstrates that exercise modulates the gut microbiota with core shared features: enhanced diversity (under specific conditions), enrichment of beneficial taxa (particularly SCFAs-producers like *Faecalibacterium*, *Roseburia*, and butyrate-producing Lachnospiraceae), increased abundance of *Akkermansia muciniphila*, and reduction in pro-inflammatory or opportunistic pathogens (e.g., Proteobacteria). This exercise-induced microbial profile is consistently associated with improved metabolic health parameters, positioning gut microbiota remodeling as a key mediator of exercise’s benefits.

## 4 Molecular mechanisms of exercise-induced gut microbiota remodeling in ameliorating PCOS

Having established the impact of exercise on gut microbiota composition, elucidating the underlying molecular mechanisms is essential to understand its therapeutic potential for PCOS. This section focuses on how exercise-induced microbial remodeling modulates metabolic networks, immune responses, and key signaling pathways to ameliorate PCOS pathology.

Exercise efficacy in PCOS management is well-supported by systematic reviews and meta-analyses (27, 75–77). Increasing evidence highlights its role in gut microbiota modulation. Clarke et al. demonstrated greater gut microbiota diversity in athletes compared to controls, a finding paralleled in animal models where six weeks of wheel running increased diversity and shifted abundances (increased Bacteroidetes, decreased Firmicutes) versus sedentary rats (78). Nevertheless, the precise molecular mechanisms linking exercise-induced microbial shifts to the reversal of core PCOS symptoms remain incompletely defined.

### 4.1 Core mechanism–systematic regulation of the gut microbiota-metabolism axis

Exercise systematically regulates gut microbiota composition and function, converging on three core mechanisms: (1) reconstruction of the SCFAs metabolic network, (2) activation of the BA-FXR signaling axis, and (3) inhibition of the LPS-inflammation axis. These interconnected pathways form a unified regulatory framework underpinning metabolic and endocrine improvements in PCOS.

#### 4.1.1 Reconstruction of the SCFAs metabolic network

Regular exercise significantly enriches Firmicutes phylum bacteria, particularly SCFAs-producing families like Ruminococcaceae, Lachnospiraceae, and Erysipelotrichaceae (74, 79, 80). Key functional examples include the enrichment of *Faecalibacterium prausnitzii* (Ruminococcaceae) in high-intensity female athletes and endurance runners, whose butyrate production promotes intestinal gluconeogenesis and glucose homeostasis (71). Similarly, Lachnospiraceae genera (*Dorea*, *Coprococcus*, *Roseburia*) demonstrate increased abundance in regular exercisers, producing acetate and butyrate to regulate blood glucose, mitigate allergic responses, and enhance gut immunity (81, 82). Marathon athletes further exhibit high enrichment of *Veillonella*, which utilizes lactate to produce SCFAs (83).

The resulting elevation in butyrate and propionate levels improves PCOS metabolic disturbances through three primary pathways. First, butyrate acts as a histone deacetylase (HDAC) inhibitor (84, 85), reducing the activity of HDAC1/2. This regulates chromatin accessibility and affects gene expression associated with processes such as androgen synthesis (e.g., CYP17A1 (32)) and gluconeogenesis. Second, propionate activates Free Fatty Acid Receptor 3 (FFAR3) on intestinal L-cells, stimulating GLP-1 secretion to enhance insulin sensitivity. GLP-1 enhances insulin secretion from pancreatic β-cells, inhibits glucagon release, delays gastric emptying, and promotes satiety, collectively contributing to improved glucose homeostasis and insulin sensitivity. Third, as the primary energy source for colonocytes, butyrate increases epithelial proliferation, upregulates tight junction proteins (ZO-1, Occludin), and reduces intestinal permeability (86, 87), preventing LPS translocation and subsequent systemic inflammation. Collectively, these SCFAs-mediated effects ameliorate metabolic dysfunction, inflammation, and barrier integrity, providing a multi-faceted foundation for PCOS symptom improvement.

The efficacy of exercise in modulating gut microbiota and metabolic health is indeed dose-dependent, with intensity serving as a critical modulator. While the term “high-intensity” applied to athletes denotes a level of training far exceeding general recommendations, the exercise intensity beneficial for PCOS management is both quantifiable and subject to an optimal range rather than a simple upper limit. Prescriptions are typically grounded in objective physiological metrics to ensure safety and efficacy. Common quantifiable measures include: (1)Percentage of Maximum Heart Rate (%HRmax): Moderate-intensity is defined as 64-76% HRmax; vigorous-intensity as 77-93% HRmax (88); (2)Percentage of Heart Rate Reserve (%HRR) or Oxygen Uptake Reserve (%VO_2_R): Moderate-intensity corresponds to 40-59% HRR/VO_2_R, while vigorous-intensity to 60-89% (89, 90); (3)Rating of Perceived Exertion (RPE): Using the Borg scale (6-20), moderate intensity targets 12-13 (“somewhat hard”), and vigorous intensity targets 14-16 (“hard” to “very hard”) (91).

For PCOS populations, evidence suggests that vigorous-intensity exercise (e.g., High-Intensity Interval Training(HIIT) protocols involving bouts at 80-90% HRmax) is highly effective for improving insulin sensitivity and cardiorespiratory fitness (76). However, the optimal intensity must be individualized. Factors such as baseline fitness, PCOS phenotype, joint health, and exercise tolerance necessitate personalization. The prevailing consensus recommends beginning with moderate-intensity exercise for sedentary individuals, progressively incorporating vigorous intervals as tolerance improves, while strictly avoiding excessive intensity that could provoke a negative stress response (92). This structured, quantifiable approach ensures that exercise intervention remains a potent, evidence-based strategy for gut microbiota and metabolic remodeling in PCOS.

#### 4.1.2 Activation of the BA-FXR signaling axis

Exercise regulates the gut-liver axis BA pool composition and function, increasing BA excretion (93, 94). By elevating the Firmicutes/Bacteroidetes ratio (55, 56, 67), exercise promotes microbial conversion of primary to secondary BAs. Exercise promotes this shift towards secondary BAs primarily by enriching gut bacteria harboring bile salt hydrolase (BSH) and 7α-dehydroxylase activities (e.g., certain Firmicutes like *Clostridium* cluster XIVa and XVI), thereby enhancing microbial conversion of primary to secondary BAs. These secondary BAs act as endogenous ligands for the FXR, recruiting nuclear receptor coactivators to activate FXR signaling with dual organ effects. The activation of FXR signaling in both liver and intestine represents a master regulator of systemic glucose and lipid metabolism, integrating microbial BA metabolism with host metabolic homeostasis. In the liver, FXR activation suppresses cholesterol 7α-hydroxylase (CYP7A1) expression to curb excessive BA synthesis while concurrently upregulating fibroblast growth factor 19 (FGF19) secretion, further inhibiting synthesis via negative feedback. Intestinally, FXR activation maintains glucose homeostasis by inhibiting hepatic gluconeogenesis enzyme expression (95) and enhances hepatic detoxification capacity. This axis provides a molecular framework for tissue-specific, multi-organ regulation of metabolic balance.

#### 4.1.3 Inhibition of the LPS-inflammation axis

Exercise reduces Proteobacteria abundance (96–98), with regular moderate-intensity resistance training specifically lowering circulating LPS levels (99). Crucially, exercise-induced increases in SCFAs, particularly butyrate, play a fundamental role in fortifying the intestinal barrier. Butyrate serves as the primary energy source for colonocytes, stimulating epithelial proliferation and upregulating tight junction proteins (ZO-1, Occludin), thereby significantly reducing intestinal permeability and preventing LPS translocation. This reduction disrupts inflammatory cascades through coordinated mechanisms. Diminished LPS impairs Toll-like receptor 4 (TLR4)/Myeloid differentiation primary response 88 (MyD88) complex formation, blocking nuclear factor kappa B (NF-κB) phosphorylation and subsequent pro-inflammatory cytokine transcription. Concurrently, SCFAs activate G-protein coupled receptor 43 (GPR43), inhibiting apoptosis-associated speck-like protein (ASC) oligomerization, caspase-1 activation, and NLRP3 inflammasome assembly. These changes collectively promote macrophage polarization from pro-inflammatory M1 to anti-inflammatory M2 phenotypes.Furthermore, SCFAs, particularly butyrate, contribute to the activation of nuclear factor erythroid 2-related factor 2 (Nrf2) by inhibiting its cytoplasmic repressor Kelch-like ECH-associated protein 1 (KEAP1). This inhibition stabilizes Nrf2 and facilitates its translocation to the nucleus, where it binds to antioxidant response elements (ARE) in the promoter regions of target genes, boosting the transcription of antioxidant enzymes (e.g., heme oxygenase-1, NAD(P)H quinone dehydrogenase 1, glutathione peroxidase) and enhancing oxidative stress resistance (100, 101).

#### 4.1.4 Integrative crosstalk and metabolic network remodeling

The three core mechanistic axes—SCFAs network reconstruction, BA-FXR signaling activation, and LPS-inflammation inhibition—do not operate in isolation but engage in extensive crosstalk, forming a dynamic and integrated metabolic network that underlies exercise’s ameliorative effects on PCOS.

SCFAs & BA/FXR: SCFAs, particularly butyrate, acting as HDAC inhibitors, may influence the epigenetic regulation of genes involved in BA metabolism (e.g., CYP7A1, FXR) and FXR target genes, potentially amplifying FXR signaling efficacy. Conversely, FXR activation in the intestine can modulate the expression of genes involved in mucosal defense and potentially influence the niche for SCFAs-producing bacteria.

SCFAs & LPS/Inflammation: Butyrate’s enhancement of gut barrier integrity is fundamental to reducing LPS translocation and systemic inflammation. Simultaneously, reduced inflammation (via LPS inhibition and SCFAs-GPR43 signaling) creates a more favorable gut environment for beneficial SCFAs-producing bacteria to thrive. SCFAs also directly suppress NLRP3 inflammasome activation via GPR43, synergizing with reduced LPS/TLR4 signaling to quell inflammation.

BA/FXR & LPS/Inflammation: Secondary BAs activating FXR can exert anti-inflammatory effects in the liver and intestine. FXR activation suppresses hepatic inflammation and may contribute to maintaining gut barrier function. Reduced systemic inflammation, in turn, may improve BA metabolism and signaling.

Convergence on Gut-Brain Axis & Central Metabolism: Key metabolites from these axes (SCFAs, secondary BAs, reduced inflammatory signals) collectively signal to the central nervous system via the gut-brain axis (vagal afferents, circulating mediators). They modulate hypothalamic neurons in the arcuate nucleus (ARC) regulating appetite (POMC/CART, AgRP/NPY), energy expenditure, and HPG axis function (kisspeptin, GnRH neurons). This central integration coordinates peripheral metabolic improvements (glucose/lipid handling, insulin sensitivity) with endocrine normalization (reduced androgens, improved gonadotropin dynamics).

This intricate crosstalk ensures that exercise-induced gut microbiota remodeling orchestrates a coordinated multi-organ response, simultaneously targeting metabolic (liver, adipose, muscle), immune (systemic, local gut, ovarian), endocrine (ovary, adrenal, HPG axis), and neural (gut-brain) systems. The resultant metabolic network remodeling—characterized by enhanced insulin sensitivity, optimized hepatic glucose/lipid output, reduced adipose tissue inflammation, suppressed ovarian androgenesis, restored central energy balance, and normalized reproductive endocrine rhythms—collectively reverses the core pathological features of PCOS.

### 4.2 Tissue-specific effects targeting the improvement of core symptoms in PCOS

Building upon the regulatory effects of exercise on metabolic and immune pathways, this section elucidates the mechanisms by which exercise-induced changes directly ameliorate the core manifestations of polycystic ovary syndrome (PCOS)—Hyperandrogenism, IR, and ovulatory dysfunction—thereby establishing a multi-targeted therapeutic framework.

#### 4.2.1 Improvement of hyperandrogenism

Hyperandrogenism pathologically characterizes an imbalance between increased androgen synthesis and reduced metabolic clearance. Exercise targets the metabolic pathways in the ovaries and liver to restore androgen homeostasis.

(1) Ovarian Targeting to Inhibit Androgen Synthesis

Exercise interventions have been shown to increase butyrate levels. Butyrate, as a histone deacetylase (HDAC) inhibitor (84, 85), has experimental evidence indicating that it can reduce histone H3K9 acetylation at the CYP17A1 promoter in relevant cell types (32), thereby blocking the transcriptional activation of this rate-limiting enzyme in androgen synthesis.

Exercise reduces plasma lipopolysaccharide (LPS) levels and diminishes LPS binding to ovarian Toll-like receptor 4 (TLR4), thereby inhibiting activation of the MyD88/NF-κB signaling axis. Consequently, this reduction alleviates the inhibitory effect of inflammatory cytokines (TNF-α, IL-6) on aromatase (CYP19A1), promoting the conversion of androgens to estrogens. Supporting this mechanism, clinical studies demonstrate that both sprint interval training and moderate-intensity continuous training significantly reduce plasma LPS and TNF-α levels after just two weeks (102).

(2) Liver Targeting to Enhance Androgen Clearance

Exercise induces the production of secondary BAs. These secondary BAs activate hepatic Farnesoid X receptor alpha (FXRα) (103), which upregulates the expression of androgen-metabolizing enzymes SULT2A1 and CYP3A4. This accelerates the sulfation and hydroxylation inactivation of testosterone.

Exercise can modulate the gut-liver axis by increasing beneficial gut bacteria and suppressing the growth of Prevotella, thereby reducing the production of bacterial β-glucuronidase. This enzyme, produced by bacteria such as Prevotella, hydrolyzes conjugated androgens back into their free, active forms. Reducing β-glucuronidase activity is crucial for limiting the enterohepatic circulation and reabsorption of androgens, thereby contributing to the regulation of systemic androgen levels.

#### 4.2.2 Alleviation of IR

Improving IR involves a synergistic interaction between peripheral metabolic regulation and central appetite suppression. Exercise targets key metabolic organs through modulation of the gut-host axis to provide effective intervention.

(1) Peripheral Metabolic Regulation in the Liver and Adipose Tissue

Exercise promotes metabolic homeostasis through the actions of secondary BAs and SCFAs on dual targets: the liver and adipose tissue. In the liver, secondary BAs activate the FXR, which directly inhibits the transcription of genes encoding phosphoenolpyruvate carboxykinase (PEPCK) and glucose-6-phosphatase (G6Pase). These enzymes are key rate-limiting factors in gluconeogenesis and glycogenolysis; their reduced activity significantly attenuates hepatic glucose production, thereby improving hyperglycemia (95). Meanwhile, in adipose tissue, SCFAs interact with the GPR43 receptor, establishing a multi-layered anti-inflammatory barrier: locally, GPR43 activation directly suppresses the assembly of the NLRP3 inflammasome, blocking the release of pro-inflammatory cytokines such as IL-1β and IL-18 (104); systemically, SCFAs regulate the migration threshold of monocytes/neutrophils and the dynamic balance between pro-inflammatory and anti-inflammatory cytokines, thereby curbing the spread of systemic inflammation (105). SCFAs and GPR43 engage in a bidirectional regulatory loop: physiological concentrations of SCFAs upregulate GPR43 expression via histone acetylation (106), and activated GPR43 further inhibits the NF-κB/MAPK signaling axis, reducing NLRP3 inflammasome activity (106). This hepatic-adipose metabolic synergy optimizes hepatic energy output via the FXR-PEPCK/G6Pase axis, while adipose tissue mitigates metabolic inflammation through the GPR43-NLRP3 pathway. Together, they form a dynamic equilibrium system maintaining “glucose-lipid metabolism-inflammation homeostasis.”

(2) Repairing the gut-brain axis to regulate central appetite

Exercise intervention enhances the production of SCFAs and BAs, regulating the gut-brain axis via a cooperative multi-receptor mechanism to maintain energy homeostasis. SCFAs activate free fatty acid receptors FFAR2/GPR43 and FFAR3/GPR41 on gut endocrine L-cells, stimulating the secretion of GLP-1 and peptide YY (PYY) (107–110). Acetate crosses the blood-brain barrier and accumulates in the hypothalamic arcuate nucleus (ARC), where it activates pro-opiomelanocortin (POMC) neurons through an FFAR2-dependent pathway (111), inhibiting agouti-related peptide (AgRP) neurons and enhancing central satiety signals. Butyrate exerts potent appetite suppression by upregulating PYY and inhibiting hypothalamic neuropeptide Y (NPY) neuron activity, largely dependent on vagus nerve-mediated FFAR3 signaling (112–114). Secondary BAs (e.g., DCA, LCA) activate TGR5 highly expressed on vagal afferents and in the ARC, suppressing AgRP/NPY neuron activity (115, 116). The BA-FXR axis further influences central metabolism via the FGF15/19 pathway (117). This integrated signaling dynamically reshapes central appetite regulation and energy homeostasis within the gut-brain axis (118, 119).

#### 4.2.3 Restoration of ovulatory function

Restoring ovulatory function involves re-establishing follicular microenvironment homeostasis and regulating endocrine rhythms. Exercise exerts beneficial effects through dual pathways involving microbiota-derived metabolites and immune modulation.

(1) Repair of the follicular microenvironment

Propionate enhances autophagy by inhibiting the NF-κB and AKT/mTOR signaling pathways and increasing LC3B protein levels. This promotes the clearance of mitochondrial damage induced by trimethylamine N-oxide (TMAO) (120), thereby helping to improve the follicular microenvironment, maintain its homeostasis, and contribute to the amelioration of ovulatory dysfunction.

Immune-Endocrine Crosstalk Regulation. Studies show that gut microbial metabolites metabolize tryptophan into aryl hydrocarbon receptor (AhR) agonists, such as indole-3-lactic acid (ILA), that activate AhR to modulate the host immune response (121). Upon binding ILA, this ligand-dependent transcription factor translocates from the cytoplasm to the nucleus and dimerizes with the AhR nuclear translocator (ARNT) to regulate downstream gene expression. AhR activation promotes the differentiation of regulatory T cells (Treg) (122). Tregs help counterbalance the activity of T helper 17 (Th17) cells, a pro-inflammatory T cell subset whose overactivation can contribute to follicular atresia. By activating AhR, ILA can suppress the differentiation and function of Th17 cells (122), thereby reducing the occurrence of follicular atresia.

(2) Regulation of endocrine rhythms

Normalization of GnRH Pulsatile Secretion. The gut microbiota influences ovarian function partly by modulating the hypothalamic-pituitary-gonadal (HPG) axis. Gut microbiota-derived metabolites, particularly SCFAs and BAs, are potent regulators of hypothalamic GnRH neuron function. Research indicates that alterations in gut microbiota composition (e.g., reductions in Firmicutes and Bacteroidetes) can increase serum levels of GLP-1 (123). GLP-1, produced by intestinal L-cells, stimulates GnRH neurons, potentially involving kisspeptin neuron intermediation. Specifically, the SCFAs (e.g., propionate) and potentially microbial-modulated BAs signal via the gut-brain axis (likely involving vagal afferents and circulating factors) to influence kisspeptin neuron activity in the hypothalamus. Kisspeptin is a critical regulator of GnRH neuron pulsatility. Exercise-induced normalization of this signaling contributes to lowering the LH/FSH ratio and restoring physiological GnRH release patterns.

Enhanced Estrogen Response. Acetate inhibits histone deacetylases (HDACs), helping to maintain normal ovarian function and follicular development, leading to elevated circulating levels of 17β-estradiol (110). Supporting this, animal studies in PCOS models demonstrate that acetate treatment significantly inhibits HDAC activity in plasma and ovaries. This inhibition counteracts the pro-inflammatory effects of TNF-α and restores nuclear factor erythroid 2–related factor 2 (Nrf2) signaling along with related antioxidant defenses (glutathione - GSH, glutathione peroxidase - GPx) (124). Furthermore, acetate administration lowers plasma testosterone levels and the LH/FSH ratio while increasing circulating 17β-estradiol and sex hormone-binding globulin (SHBG) levels in these models (124). Collectively, acetate’s HDAC inhibition contributes to improved ovarian steroidogenesis and estrogen response.

**Figure 4:**
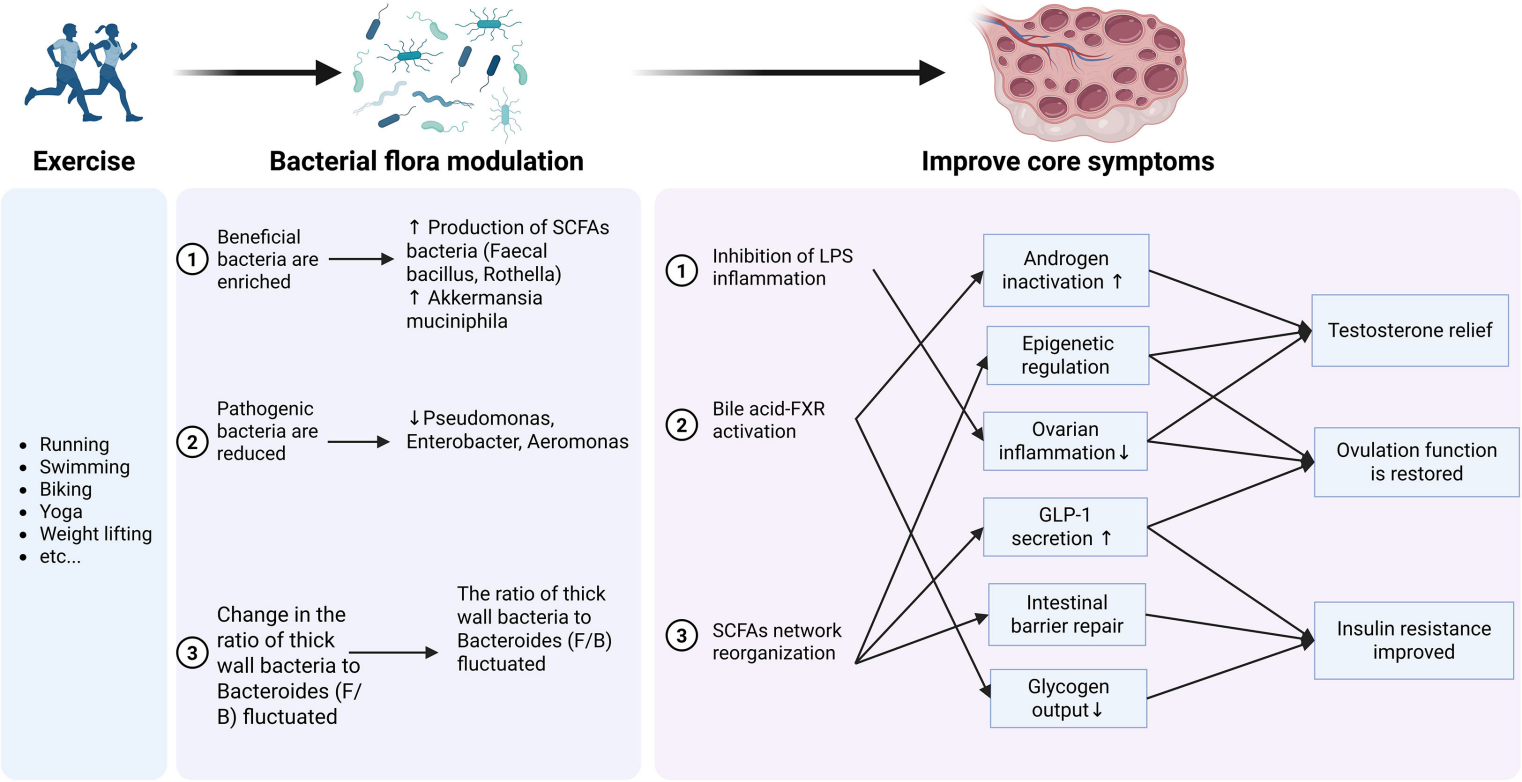
Mechanisms by which exercise ameliorates PCOS through remodeling the gut microbiota.

This figure (**Figure 4**) summarizes the core mechanisms through which exercise-induced changes in the gut microbiota improve the core symptoms of PCOS. These include the reorganization of the SCFAs network, activation of the BA-FXR signaling axis, and inhibition of the LPS-inflammation axis, which collectively target hyperandrogenism, insulin resistance, and ovulatory dysfunction in a tissue-specific manner.

### 4.3 Ecological basis of exercise-driven microbial response and its implications for specificity

The interaction between exercise and the gut microbiome is far more than a simple change in composition. Exercise can induce physiological changes, reshape the gut environment, and drive microbial community restructuring.

#### 4.3.1 Driving factors

Alterations in Gut Oxygen Supply: Exercise, particularly endurance training, improves cardiovascular function and may temporarily increase gut perfusion and oxygenation (125). This favors the expansion of facultative anaerobes (e.g., certain *Lactobacillus* spp.) and obligate aerobes, while potentially limiting strictly anaerobic bacteria, including many SCFAs producers. However, the net effect of exercise is generally an increase in SCFAs producers, suggesting the presence of compensatory mechanisms or ecological niche adaptation (78).

BA Flow and Composition: Exercise enhances the excretion of BAs (86, 87) and regulates the gut-liver axis. Changes in the BA profile (e.g., increased secondary BAs) can specifically inhibit or promote the growth of certain bacteria that are sensitive to these antimicrobial molecules (42, 93).

Immune System Regulation: Exercise alters both systemic and mucosal immunity. The release of exercise-induced myokines (e.g., IL-6) and changes in immunoglobulin A (IgA) secretion can influence microbial growth and adhesion (54).

#### 4.3.2 Differential impact of exercise modalities on PCOS subtypes

The heterogeneity of PCOS is well-recognized and is formally categorized into phenotypic subtypes based on the Rotterdam diagnostic criteria, which require the presence of at least two of the following three features: (1) hyperandrogenism (clinical and/or biochemical), (2) ovulatory dysfunction, and (3) polycystic ovarian morphology on ultrasound (126). This yields four distinct phenotypes: (1)Phenotype A (classic PCOS): Hyperandrogenism + Ovulatory dysfunction + Polycystic ovaries; (2)Phenotype B: Hyperandrogenism + Ovulatory dysfunction; (3)Phenotype C: Hyperandrogenism + Polycystic ovaries; (4)Phenotype D: Ovulatory dysfunction + Polycystic ovaries (non-hyperandrogenic). Beyond this phenotypic classification, stratification by the presence of obesity (e.g., BMI ≥25 kg/m² vs. lean) and the severity of insulin resistance (e.g., HOMA-IR >2.1 or based on glucose clamp studies) is crucial for understanding metabolic risk and tailoring therapy (127, 128). It is within this framework of phenotypic and metabolic heterogeneity that the differential effects of exercise modalities must be considered.

Given the heterogeneity of polycystic ovary syndrome (PCOS) (e.g., obesity vs. lean, IR-dominant vs. hyperandrogenism-dominant), and the unique physiological effects of different types of exercise, specific interventions for certain subtypes may be more effective. Critically, these exercise modalities engage distinct metabolic pathways, which underpins their differential therapeutic effects.

Endurance/Aerobic Training: This modality primarily enhances cardiovascular health and insulin sensitivity through AMPK activation and improved mitochondrial biogenesis in skeletal muscle. Its significant effects on increasing SCFAs producers (e.g., *Faecalibacterium, Roseburia*) and reducing systemic inflammation (72, 74, 102) may be especially beneficial for PCOS women with IR and metabolic dysfunction, regardless of obesity status. The potential increase in *Akkermansia muciniphila* (73) also supports metabolic health.

Resistance/Strength Training: This is effective in increasing muscle mass, improving basal metabolic rate, and glucose handling capacity primarily via mTORC1-mediated muscle protein synthesis. Its documented ability to reduce opportunistic pathogens (e.g., *Pseudomonas, Aeromonas*) and circulating LPS levels (68, 74) may offer an advantage for PCOS women with significant chronic low-grade inflammation and androgen excess, particularly in the context of obesity, where inflammation is often exacerbated.

HIIT: HIIT provides time-efficient improvements in metabolic health and cardiovascular function by stimulating both aerobic and anaerobic systems, leading to exaggerated post-exercise oxygen consumption (EPOC) and enhanced GLUT4 translocation. Although studies specifically targeting the relationship between PCOS and gut microbiome changes with HIIT are limited, its significant effects on insulin sensitivity and reduction of androgens (76) suggest it may be beneficial for various subtypes, though its intensity must be carefully personalized based on individual tolerance.

Understanding these ecological driving factors and the distinct microbial and metabolic signatures induced by specific exercise patterns (e.g., HIIT’s rapid metabolic improvement linked to specific SCFAs producers; resistance training’s anti-inflammatory effect via pathogen reduction) provides a molecular rationale for designing personalized exercise prescriptions. These prescriptions can be tailored to target the primary pathological drivers in different PCOS phenotypes: Endurance/aerobic training may be prioritized for IR-dominant phenotypes (regardless of BMI) due to its potent effects on SCFAs producers and systemic inflammation. Resistance training might offer advantages for obese PCOS women with significant chronic inflammation and androgen excess by reducing LPS and opportunistic pathogens. HIIT’s efficiency in improving insulin sensitivity and reducing androgens suggests broad applicability, but intensity should be personalized.

Furthermore, the efficacy of exercise is modulated by dietary patterns, creating a critical exercise-diet interaction that shapes the gut microbiota. For instance, the enrichment of SCFA-producing bacteria (e.g., Faecalibacterium) through exercise can be synergistically amplified by a high-fiber, prebiotic-rich diet, which provides essential substrates for microbial fermentation (129). Conversely, the benefits of exercise may be diminished by a Western-style diet high in saturated fats and refined sugars, which promotes dysbiosis and inflammation. Therefore, combining tailored exercise protocols with evidence-based dietary interventions (e.g., Mediterranean or low-glycemic index diets) represents a most promising strategy for optimizing gut microbiome remodeling and achieving sustainable metabolic and endocrine improvements in PCOS (130, 131).

It is noteworthy that the beneficial role of exercise presents a dose-dependent characteristic. This review focuses on the mechanisms by which moderate exercise ameliorates PCOS; however, it is observed in clinical practice that prolonged high-intensity exercise, often coupled with psychological stress (e.g., in some athletes), can conversely lead to endocrine dysfunction and menstrual disturbances. This apparent paradox highlights the critical importance of exercise intensity and individual tolerance. The underlying mechanism may involve excessive activation of the hypothalamic-pituitary-adrenal (HPA) axis, leading to elevated cortisol levels that suppress GnRH pulsatility and ovarian function (132), while also potentially exacerbating gut dysbiosis and inflammation, thereby counteracting the benefits brought by moderate exercise. Consequently, the exercise prescriptions recommended for PCOS management should be tailored to the individual, aiming to achieve the beneficial “eustress” that remodels the gut microbiota and metabolism, while avoiding excessive intensity that leads to detrimental “distress”.

## 5 Conclusion

In conclusion, this review synthesizes evidence establishing exercise-induced gut microbiota remodeling as a key mechanism ameliorating PCOS pathology. Up-to-date evidence demonstrates that exercise enriches beneficial taxa (e.g., SCFA-producing Faecalibacterium, Roseburia, Akkermansia) while suppressing pro-inflammatory pathogens (e.g., Proteobacteria), thereby elevating beneficial metabolites (SCFAs, secondary BAs) and reducing detrimental factors (LPS). These microbial shifts activate a coordinated molecular response through key receptors (FXR, GPR43, TGR5, AhR) and inhibit critical pathogenic pathways (TLR4/NF-κB, NLRP3, HDAC), culminating in a multi-organ reversal of PCOS features: improved insulin sensitivity and metabolic homeostasis, suppressed hyperandrogenism, restored ovulatory function, and resolved chronic inflammation.

Despite this compelling framework, significant limitations remain. Most mechanistic insights are derived from animal models, lacking robust translational human data. The profound heterogeneity of PCOS is often overlooked, and the optimal exercise protocol (intensity, frequency, duration) remains undefined. Furthermore, the long-term sustainability of exercise-induced microbial and symptomatic benefits is unclear.

Future research must prioritize human studies incorporating multi-omics approaches and interventional designs like FMT to establish causality. Leveraging gut microbial signatures and metabolite profiles as biomarkers will be crucial for developing personalized exercise prescriptions tailored to PCOS phenotypes. Ultimately, translating these mechanistic insights into microbiome-targeted therapies—whether through prebiotics, microbial metabolites, or their analogs—holds exceptional promise for revolutionizing the management of PCOS.

## Glossary

AhR: Aryl Hydrocarbon Receptor
AMPK: AMP-activated Protein Kinase
BA: Bile Acid
BCAA: Branched-Chain Amino Acid
BSH: Bile Salt Hydrolase
FXR: Farnesoid X Receptor
GLP-1: Glucagon-Like Peptide-1
GnRH: Gonadotropin-Releasing Hormone
GPR43/FFAR3: G Protein-Coupled Receptor 43/Free Fatty Acid Receptor 3;HDAC, Histone Deacetylase
HPG: Hypothalamic-Pituitary-Gonadal Axis
IR: Insulin Resistance
LPS: Lipopolysaccharide;mTORC1, Mechanistic Target of Rapamycin Complex 1
NF-κB: Nuclear Factor Kappa B
NLRP3: NLR Family Pyrin Domain Containing 3
PCOS: Polycystic Ovary Syndrome
SCFAs: Short-Chain Fatty Acid
TLR4: Toll-Like Receptor 4
TMAO: Trimethylamine N-Oxide
A. muciniphila: *Akkermansia muciniphila*
F/B ratio: Firmicutes/Bacteroidetes Ratio
F. prausnitzii: *Faecalibacterium prausnitzii*
CYP17A1: 17α-Hydroxylase
CYP19A1: Aromatase
FGF19: Fibroblast Growth Factor 19
G6Pase: Glucose-6-Phosphatase
PEPCK: Phosphoenolpyruvate Carboxykinase
SULT2A1: Sulfotransferase 2A1
IL-6/IL-1β/TNF-α: Interleukin-6/1β/Tumor Necrosis Factor-α
Th17/Treg: T Helper 17/Regulatory T Cell
TLR4/MyD88: Toll-Like Receptor 4/Myeloid Differentiation Primary Response 88
HIIT: High-Intensity Interval Training
VWR: Voluntary Wheel Running
FMT: Fecal Microbiota Transplantation
ROS: Reactive Oxygen Species
SHBG: Sex Hormone-Binding Globulin.
